# In this Issue

**Published:** 2013

## ALCOHOL METABOLISM AND EPIGENETIC CHANGES

Alcohol can influence gene expression, and specifically epigenetic regulatory processes that modify the activity of genes, through a variety of mechanisms. Some of these are related to the metabolism of alcohol in the cells, explains Dr. Samir Zakhari. In general, several metabolites, such as nicotinamide-adenine dinucleotide (NAD) in its oxidized and reduced forms, acetyl-coenzyme A (acetyl-CoA), and S-adenosylmethionine (SAM) serve as cofactors for numerous reactions in the cell, including reactions related to epigenetic DNA and histone modifications. Dr. Zakhari reviews some of the pathways through which alcohol metabolism alters the levels of these metabolites in the cells and how the changes in metabolite levels may impact epigenetic processes such as DNA methylation and histone acetylation. (pp. 6–16)

## DYSREGULATION OF microRNA EXPRESSION AND FUNCTION CONTRIBUTES TO THE ETIOLOGY OF FETAL ALCOHOL SPECTRUM DISORDERS

MicroRNAs (miRNAs) are small, noncoding RNA molecules that help mediate epigenetic regulatory mechanisms. Recent research has indicated that these miRNAs also are targets of ethanol’s actions, particularly during fetal development. As Dr. Sridevi Balaraman, Mr. Joseph D. Tingling, Mr. Pai-Chi Tsai, and Dr. Rajesh C. Miranda explain, alcohol-induced dysregulation of miRNAs likely is involved in the etiology of fetal alcohol spectrum disorders (FASD). The authors summarize the current knowledge on how alcohol interferes with the formation, cellular localization, and function of miRNAs and the implications of this interference for the development as well as the management of FASD. (pp. 18–24)

## ALCOHOL, DNA METHYLATION, AND CANCER

Chronic drinking is a strong risk factor for several types of cancer, particularly of the upper aerodigestive tract, liver, colorectum, and breast. According to Drs. Marta Varela-Rey, Ashwin Woodhoo, Maria-Luz Martinez-Chantar, José M. Mato, and Shelly C. Lu, the role of epigenetic mechanisms, such as DNA methylation, in alcohol-induced carcinogenesis is increasingly being recognized. In particular, chronic alcohol exposure may affect the pathways that regulate the availability of S-adenosylmethionine, the principal biological methyl donor for methylation reactions. Additionally, alcohol may interfere with the activities of various enzymes involved in DNA methylation. All of these mechanisms may contribute to both the initiation and the progression of alcohol-induced tumors. (pp. 25–35)

## IN UTERO ALCOHOL EXPOSURE, EPIGENETIC CHANGES, AND THEIR CONSEQUENCES

Prenatal alcohol exposure can lead to detrimental effects collectively known as fetal alcohol spectrum disorders (FASD). Alcohol’s effects on the developing brain during critical periods of differentiation and growth are particularly devastating and can result in cognitive and behavioral deficits. As Ms. Michelle Ungerer, Mr. Jaysen Knezovich, and Dr. Michele Ramsay explain, interference with normal epigenetic mechanisms likely is an important component of alcohol’s harmful effects on the developing fetus. To study these effects, researchers have to rely strongly on animal models that allow them to control numerous variables, such as the specific timing, duration, and level of alcohol exposure. The authors summarize the findings of such studies to date and discuss how epigenetic mechanisms may be involved in developmental reprogramming, the effects of preconception alcohol exposure, and the transgenerational transmission of effects of alcohol exposure. (pp. 37–46)

## EPIGENETIC EFFECTS OF ETHANOL ON THE LIVER AND GASTROINTESTINAL SYSTEM

Alcohol has been shown to interfere with several epigenetic mechanisms in the gastrointestinal tract and liver, including DNA methylation, site-specific histone modifications, and microRNAs. In this article, Drs. Shivendra D. Shukla and Robert W. Lim discuss the mechanisms through which alcohol exposure can affect epigenetic processes and explain how these disruptions can contribute to such alcohol-related disorders as fatty liver and liver cancer. The findings to date also suggest that alcohol’s epigenetic effects on the gastrointestinal tract and liver may contribute to additional pathophysiological responses to alcohol in multiple other organs. (pp. 47–55)

## EPIGENETIC EVENTS IN LIVER CANCER RESULTING FROM ALCOHOLIC LIVER DISEASE

Alcoholic liver disease (ALD) in some cases can result in the development of liver cancer. Epigenetic mechanisms play a crucial role in this process, according to Dr. Samuel W. French. For example, DNA methylation and histone methylation and acetylation can promote the reversion of normal liver cells into progenitor and stem cells whose unchecked growth is involved in liver cancer development. Dr. French describes how chronic alcohol consumption can alter normal epigenetic patterns through a variety of mechanisms, including products of ethanol metabolism, effects on signaling pathways, and indirect actions via other regulatory molecules. All of these pathways can play a role in alcohol-associated liver cancer pathogenesis. (pp. 57–67)

## EPIGENETIC CONTROL OF GENE EXPRESSION IN THE ALCOHOLIC BRAIN

Chronic alcohol exposure affects gene expression throughout the body, but its effects on brain gene expression are particularly important because they contribute to cellular adaptations that ultimately lead to behavioral tolerance and alcohol dependence. One mechanism through which alcohol induces changes in brain gene expression involves alterations in various epigenetic regulatory processes, reports Dr. Igor Ponomarev. These alterations, particularly in the actions of certain epigenetic “master regulators” can affect various types of brain cells and their function and thereby play a crucial role in the development of alcohol addiction. (pp. 69–76)

## PRENATAL ALCOHOL EXPOSURE AND CELLULAR DIFFERENTIATION: A ROLE FOR POLYCOMB AND TRITHORAX GROUP PROTEINS IN FAS PHENOTYPES?

Emerging research suggests that ethanol can impair fetal physical, behavioral, and cognitive development, resulting in such conditions as fetal alcohol spectrum disorders (FASD) and its most devastating form, fetal alcohol syndrome. Alcohol exerts its detrimental effects by interfering with the execution of molecular programs governing differentiation. For example, ethanol exposure disrupts cellular migration, changes cell–cell interactions, and alters growth factor signaling pathways, as well as other epigenetic mechanisms controlling gene expression. In this article, Ms. Kylee J. Veazey, Ms. Daria Muller, and Dr. Michael C. Golding discuss the consequences of alcohol exposure on fetal development, focusing on two crucial protein complexes (i.e., the Polycomb and Trithorax proteins) and their role in the etiology of FASD. (pp. 77–85)

## CIRCADIAN DISRUPTION: POTENTIAL IMPLICATIONS IN INFLAMMATORY AND METABOLIC DISEASES ASSOCIATED WITH ALCOHOL

Circadian rhythms are a prominent and critical feature of cells, tissues, organs, and behavior that help an organism function most efficiently and anticipate things such as food availability. Therefore, it is not surprising that disrupted circadian rhythms or misalignment between central and peripheral circadian rhythms predispose to and/or exacerbate a wide variety of diseases, including alcohol-associated disorders. This article by Drs. Robin M. Voigt, Christopher B. Forsyth, and Ali Keshavarzian discusses the interplay between alcohol consumption and circadian-rhythmicity and explores how circadian-rhythm disruption affects immune function and metabolism. It also summarizes potential epigenetic mechanisms that may be contributing to this phenomenon. (pp. 87–96)

## EPIGENETIC TARGETS FOR REVERSING IMMUNE DEFECTS CAUSED BY ALCOHOL EXPOSURE

Chronic heavy drinking can weaken the immune system, thereby increasing the risk of medical conditions related to immune system dysfunction (e.g., acute respiratory distress syndrome, liver cancer, and alcoholic liver disease), enhancing susceptibility to many infections, and accelerating progression of HIV infection. According to Drs. Brenda J. Curtis, Anita Zahs, and Elizabeth J. Kovacs, epigenetic mechanisms play a pivotal role in these processes. Thus, epigenetic modifications can promote exaggerated inflammatory responses through a variety of mechanisms as well as interfere with the body’s defense against invasion by harmful micro-organisms in the gut and respiratory system. The authors also discuss how a better understanding of alcohol’s effects on epigenetic mechanisms may lead to promising new treatment approaches. (pp. 97–113)

